# *In Silico* Identification of Functional Protein Interfaces

**DOI:** 10.1002/cfg.309

**Published:** 2003-07

**Authors:** Rachel E. Bell, Nir Ben-Tal

**Affiliations:** Department of Biochemistry The George S. Wise Faculty of Life Sciences Tel Aviv University Ramat Aviv 69978 Israel

## Abstract

Proteins perform many of their biological roles through protein–protein, protein–DNA or protein–ligand interfaces. The identification of the amino acids comprising these interfaces often enhances our understanding of the biological function of the proteins. Many methods for the detection of functional interfaces have been developed, and large-scale analyses have provided assessments of their accuracy. Among them are those that consider the size of the protein interface, its amino acid composition and its physicochemical and geometrical properties. Other methods to this effect use statistical potential functions of pairwise interactions, and evolutionary information. The rationale of the evolutionary approach is that functional and structural constraints impose selective pressure; hence, biologically important interfaces often evolve at a slower pace than do other external regions of the protein. Recently, an algorithm, Rate4Site, and a web-server, ConSurf (http://consurf.tau.ac.il/), for the identification of functional interfaces based on the evolutionary relations among homologous proteins as reflected in phylogenetic trees, were developed in our laboratory. The explicit use of the tree topology and branch lengths makes the method remarkably accurate and sensitive. Here we demonstrate its potency in the identification of the functional interfaces of a hypothetical protein, the structure of which was determined as part of the international structural genomics effort. Finally, we propose to combine complementary procedures, in order to enhance the overall performance of methods for the identification of functional interfaces in proteins.


**Comparative and Functional Genomics**
*Comp Funct Genom* 2003; **4**: 420–423.
Published online in Wiley InterScience (www.interscience.wiley.com). **DOI:** 10.1002/cfg.309

**Conference Review**

### *In silico* identification of functional protein interfaces

Rachel E. Bell and Nir Ben-Tal*
*Department of Biochemistry, The George S. Wise Faculty of Life Sciences, Tel Aviv University, Ramat Aviv 69978, Israel*

**Correspondence to:*
*Nir Ben-Tal, Department of Biochemistry, The George S. Wise Faculty of Life Sciences, Tel Aviv University, Ramat Aviv 69978, Israel.*
*E-mail: bental@ashtoret.tau.ac.il;*
*Web: http://ashtoret.tau.ac.il/*

Received: 22 May 2003
Revised: 3 June 2003
Accepted: 3 June 2003

### Abstract

**Proteins perform many of their biological roles through protein–protein, protein–DNA or protein–ligand interfaces. The identification of the amino acids comprising these interfaces often enhances our understanding of the biological function of the proteins. Many methods for the detection of functional interfaces have been developed, and large-scale analyses have provided assessments of their accuracy. Among them are those that consider the size of the protein interface, its amino acid composition and its physicochemical and geometrical properties. Other methods to this effect use statistical potential functions of pairwise interactions, and evolutionary information. The rationale of the evolutionary approach is that functional and structural constraints impose selective pressure; hence, biologically important interfaces often evolve at a slower pace than do other external regions of the protein. Recently, an algorithm, Rate4Site, and a web-server, ConSurf (http://consurf.tau.ac.il/), for the identification of functional interfaces based on the evolutionary relations among homologous proteins as reflected in phylogenetic trees, were developed in our laboratory. The explicit use of the tree topology and branch lengths makes the method remarkably accurate and sensitive. Here we demonstrate its potency in the identification of the functional interfaces of a hypothetical protein, the structure of which was determined as part of the international structural genomics effort. Finally, we propose to combine complementary procedures, in order to enhance the overall performance of methods for the identification of functional interfaces in proteins. Copyright © 2003 John Wiley & Sons, Ltd.**

**Keywords:** **functional regions; inter-protein interfaces; evolutionary relations; evolutionary rate; evolutionary conservation; homologous proteins**

### Introduction

The discrimination of functional oligomeric protein–protein interfaces observed in three-dimensional (3D) structures from contacts that are artifacts of crystallization is a challenging question. Many criteria have previously been used to identify and characterize protein–protein interfaces. These rely on considerations of the solvent accessible surface area buried upon association [14,13]; free energy changes upon alanine-scanning mutations [31]; experimental [35] and *in silico* [25] two-hybrid systems; scoring functions based on statistical potentials [21,26]; and physicochemical and geometric properties of the surface, such as electrostatics [28], hydrophobicity [32], amino acid composition [22] and shape complementarity or planarity [18,15,12]. Other approaches are based on scoring evolutionary conservation. The simplest of these are based on estimating the local amino acid's information content or 'relative entropy' [7,34] and the more advanced involve phylogenetic reconstruction [17]. Other forms of utilizing phylogenetic information to this effect include the tracing of correlated mutations [23] and similarities between phylogenetic trees [24].

The most rudimentary approach for the identification of biological inter-protein interfaces uses

Copyright © 2003 John Wiley & Sons, Ltd.

the solvent accessibility of the protein surface area (ASA). This approach is based on two fundamental assumptions: (a) biologically significant interfaces must be thermodynamically stable; and (b) relative stability is, by and large, proportional to the number of interacting atoms. Thus, the larger the surface area buried upon association, the more likely the interface is to be biologically relevant. Ponstingl *et al.* [26] showed that ASA correctly classified homodimers and monomers in 85% of cases.

In cases where a sufficient number of homologous proteins are available, evolutionary-based methods may aid in the discrimination between biological and non-biological contacts. Elcock and McCammon [7] showed that a simple relative entropy criterion can be used to correctly discriminate between X-ray crystal interfaces observed for homodimeric and monomeric proteins in 86% of cases. Valdar and Thornton [34] combined the ASA and conservation measures using statistical tests and accurately identified 92% of the examined inter-protein interfaces. Such methods are also commonly used for the identification of other functional interfaces involved, e.g. in catalytic activity and ligand-, peptide- or DNA-binding. Numerous applications using different evolutionary-based methods have been developed to this effect [1,3,4,6–9,11,16,19,29]. For example, Madabushi *et al.* [20] and Yao *et al.* [36] applied the Evolutionary Trace (ET) method for the identification of various known functional interfaces, and reported a success rate of 90% or more.

Valdar [33] presented a critical review of 19 different methods for the scoring of evolutionary conservation, including the ET method. The conclusion was that none of the methods achieved statistical or biological rigour, presumably since they all suffer from inadequate treatment of the evolutionary process. The Rate4Site algorithm [27], which was recently developed in our laboratory and was not reviewed by Valdar, provides a more accurate treatment of the process. Rate4Site accepts as input a phylogenetic tree reconstructed from a multiple-alignment of the homologous sequences, and provides a maximum likelihood estimate of the evolutionary rates of the amino acid sites. The topology and branch lengths of the tree, as well as the underlying stochastic process of the evolution of the homologues, are explicitly taken into account in the calculations, which makes Rate4Site very accurate and sensitive; we demonstrated that the algorithm is superior to other methods in the identification of various functional regions [27]. We have also developed a web-server, ConSurf, which implements this algorithm for proteins with a known 3D-structure (**http://consurf.tau.ac.il/** [10]).

Recently, a large-scale analysis was carried out using the ET method in combination with several statistical-significance tests, in an attempt to identify known functional interfaces in a set of 86 proteins [36]. With a few exceptions, the method correctly identified these interfaces, further demonstrating the power of evolutionary-based methods. Here we demonstrate that ConSurf was sensitive enough to identify the ATP binding-pocket of putative protein Mj0577, which was overlooked in the ET analysis.

### Methods

For comparison, we attempted to use an input that is as similar as possible to the one that was generated by Yao *et al.* [36]. The reference structure (PDB code: 1mjh [37]) was entered into the ConSurf web-server; 19 homologues were retrieved from the SWISSPROT database [5], using PSI–BLAST ( [2]; E-score threshold of 0.05), and aligned using CLUSTAL W [30]. The alignment is available at: **http://ashtoret.tau.ac.il/~rebell**.

### Results and discussion

One of the major problems with hypothetical proteins is how to relate them to known protein families. In the case of the putative protein Mj0577 the homologues are also classified as hypothetical; furthermore, the sequence identity among the homologous proteins can be as low as ~15%. We demonstrate here that ConSurf successfully detected the ATP binding-groove of the protein (Figure 1). The residues comprising the ATP binding-site are not strictly conserved among the homologues, which may be the reason why the ET analysis of Yao *et al.* [36] failed to significantly detect it. Nevertheless, ConSurf assigns these residues with reasonably high conservation scores. Interestingly, the ConSurf analysis also shows that the homodimer–protein interface, which was observed in the X-ray crystal structure and was not examined by

Copyright © 2003 John Wiley & Sons, Ltd.

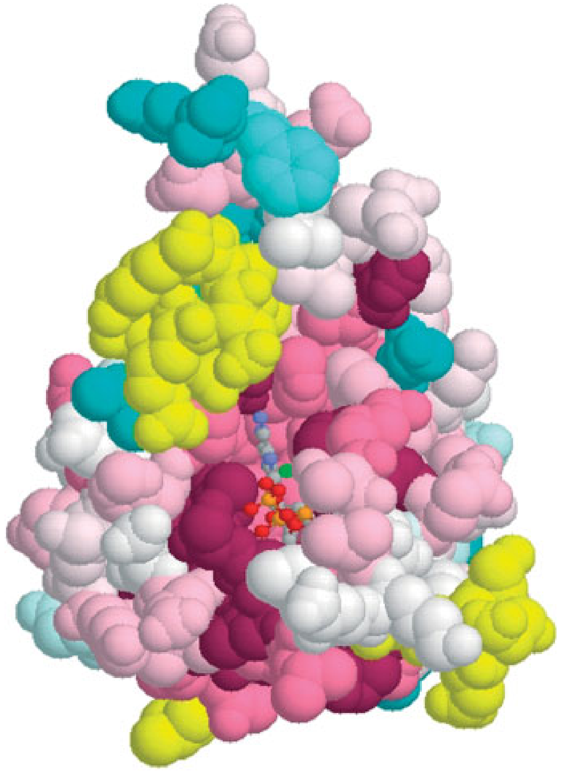

**Figure 1.** The conserved ATP-binding groove in the ATP-binding domain of hypothetical protein Mj0577 (PDB code: 1mjh [37]). The domain is represented as a space-filled model, and the ATP and Mn ion are shown as ball-and-stick models. The evolutionary rates are colour-coded onto the domain as follows: slowly evolving residues are maroon, residues that evolve at average rates are white, and rapidly evolving residues are turquoise. Residues aligned with less than 10 sequences out of a total of 19 and whose conservation grades are of low confidence are marked in yellow. Several amino acids that are directly involved in ATP-binding (Asp13, Val41, Gly127, Gly130, Gly140, Ser141, Val142 and Thr143) receive the highest conservation grades; yet none of them is exclusively invariable among the homologous proteins. They interact with the ATP molecule mainly through their backbone

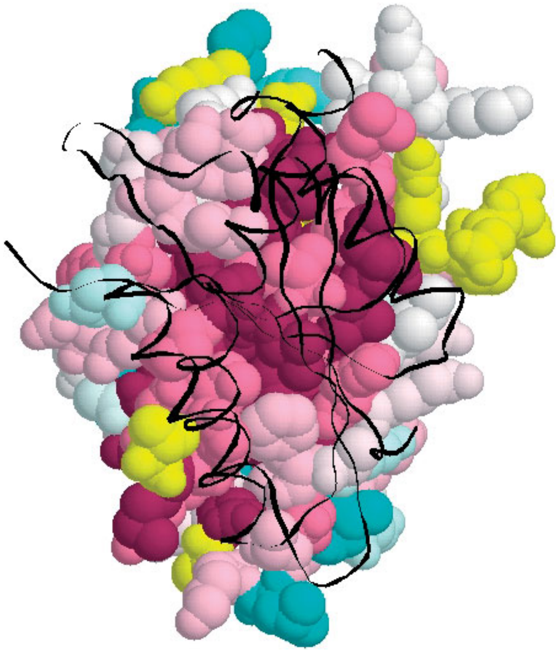

**Figure 2.** The conserved homodimer interface of the ATP-binding domain of the hypothetical protein Mj0577. The evolutionary rates are colour-coded and mapped onto the space-filled representation of one of the monomers using the colour scheme of Figure 1; the counterpart monomer is shown as black ribbons

Yao *et al.*, is highly conserved, suggesting that this interface is biologically important (Figure 2).

Recent progress in structure determination techniques has resulted in a major increase in the number of novel high-resolution 3D structures with unknown function. Evolutionary analysis of these proteins can provide means for the identification of their functional regions. We have analysed dozens of proteins with unknown function (data not shown). The bottleneck in the identification of the functional interfaces in such proteins appears to be the number of putatively related homologues and their degree of similarity. Methods that make use of evolutionary information require a minimum number of homologous proteins to provide a sufficient span of their evolutionary history. Our experience has been that even ConSurf, in spite of its high accuracy and sensitivity, is somewhat limited when dealing with less than 10 homologous proteins. It is also noteworthy to recall that some rather important functional interfaces are not evolutionarily conserved; the hyper-variable peptide recognition groove in MHC molecules is an excellent example of this. In cases where there is an insufficient number of homologues, some of the complementary methods mentioned above may be used.

### Acknowledgements

We are grateful to the Bioinformatics Unit at the George S. Wise Faculty of Life Sciences at Tel Aviv University for providing technical assistance and computational facilities, and to Karen B. Avraham for her assistance. This study was supported by a Research Career Development Award from the Israel Cancer Research Fund.

Copyright © 2003 John Wiley & Sons, Ltd. 

### References

1. Aloy P, Querol E, Aviles FX, Sternberg MJ. 2001. Automated structure-based prediction of functional sites in proteins: applications to assessing the validity of inheriting protein function from homology in genome annotation and to protein docking. *J Mol Biol* **311**: 395–408.
2. Altschul SF,, Madden TL, Schäffer AA, *et al*. 1997. Gapped BLAST and PSI–BLAST: a new generation of protein database search programs. *Nucleic Acids Res* **25**: 3389–3402.
3. Armon A, Graur D, Ben-Tal N. 2001. ConSurf: an algorithmic tool for the identification of functional regions in proteins by surface mapping of phylogenetic information. *J Mol Biol* **307**: 447–463.
4. Blouin CY, Boucher Y, Roger AJ. 2003. Inferring functional constraints and divergence in protein families using 3D mapping of phylogenetic information. *Nucleic Acids Res* **31**: 790–797.
5. Boeckmann B, Bairoch A, Apweiler R, *et al*. 2003. The SWISS-PROT protein knowledge base and its supplement TrEMBL in 2003. *Nucleic Acids Res* **31**: 365–370.
6. Dean AM, Golding GB. 2000. Enzyme evolution explained sort of. *Pac Symp Biocomput* 6–17.
7. Elcock AH, McCammon JA. 2001. Identification of protein oligomerization states by analysis of interface conservation. *Proc Natl Acad Sci USA* **98**: 2990–2994.
8. Fleming MA, Potter JD, Ramirez CJ, Ostrander GK, Ostrander EA. 2003. Understanding missense mutations in the BRCA1 gene: an evolutionary approach. *Proc Natl Acad Sci USA* **100**: 1151–1156.
9. Gaucher EA, Miyamoto MM, Benner SA. 2001. Function–structure analysis of proteins using covarion-based evolutionary approaches: elongation factors. *Proc Natl Acad Sci USA* **98**: 548–552.
10. Glaser F, Pupko T, Paz I, *et al*. 2003. ConSurf: identification of functional regions in proteins by surface-mapping of phylogenetic information. *Bioinformatics* **19**: 163–164.
11. Henikoff S, Henikoff JG. 1991. Automated assembly of protein blocks for database searching. *Nucleic Acids Res* **19**: 6565–6572.
12. Hu Z, Ma B, Wolfson H, Nussinov R. 2000. Conservation of polar residues as hot spots at protein interfaces. *Proteins* **39**: 331–342.
13. Janin J. 1997. Specific vs. non-specific contacts in protein crystals. *Nature Struct Biol* **4**: 973–974.
14. Janin J, Rodier F. 1995. Protein–protein interaction at crystal contacts. *Proteins* **23**: 580–587.
15. Jones S, Thornton JM. 1997. Analysis of protein–protein interaction sites using surface patches. *J Mol Biol* **272**: 121–132.
16. Landgraf R, Xenarios I, Eisenberg D. 2001. Three-dimensional cluster analysis identifies interfaces and functional residue clusters in proteins. *J Mol Biol* **307**: 1487–1502.
17. Lichtarge O, Sowa ME. 2002. Evolutionary predictions of binding surfaces and interactions. *Curr Opin Struct Biol* **12**: 21–27.
18. Lijnzaad P, Argos P. 1997. Hydrophobic patches on protein subunit interfaces: characteristics and prediction. *Proteins* **28**: 333–343.
19. Lockless SW, Ranganathan R. 1999. Evolutionarily conserved pathways of energetic connectivity in protein families. *Science* **286**: 295–299.
20. Madabushi S, Yao H, Marsh M, *et al*. 2002. Structural clusters of evolutionary trace residues are statistically significant and common in proteins. *J Mol Biol* **316**: 139–154.
21. Moont G, Gabb HA, Sternberg MJ. 1999. Use of pair potentials across protein interfaces in screening predicted docked complexes. *Proteins* **35**: 364–373.
22. Ofran Y, Rost B. 2003. Analysing six types of protein–protein interfaces. *J Mol Biol* **325**: 377–387.
23. Pazos F, Helmer-Citterich M, Ausiello G, Valencia A. 1997. Correlated mutations contain information about protein–protein interaction. *J Mol Biol* **271**: 511–523.
24. Pazos F, Valencia A. 2001. Similarity of phylogenetic trees as indicator of protein–protein interaction. *Protein Eng* **14**: 609–614.
25. Pazos F, Valencia A. 2002. *In silico* two-hybrid system for the selection of physically interacting protein pairs. *Proteins* **47**: 219–227.
26. Ponstingl H, Henrick K, Thornton JM. 2000. Discriminating between homodimeric and monomeric proteins in the crystalline state. *Proteins* **41**: 47–57.
27. Pupko T, Bell RE, Mayrose I, Glaser F, Ben-Tal N. 2002. Rate4Site: an algorithmic tool for the identification of functional regions in proteins by surface mapping of evolutionary determinants within their homologues. *Bioinformatics* **18**(suppl 1): S71–77.
28. Sheinerman FB, Norel R, Honig B. 2000. Electrostatic aspects of protein–protein interactions. *Curr Opin Struct Biol* **10**: 153–159.
29. Simon AL, Stone EA, Sidow A. 2002. Inference of functional regions in proteins by quantification of evolutionary constraints. *Proc Natl Acad Sci USA* **99**: 2912–2917.
30. Thompson JD, Higgins DG, Gibson TJ. 1994. CLUSTAL W: improving the sensitivity of progressive multiple sequence alignment through sequence weighting, position-specific gap penalties and weight matrix choice. *Nucleic Acids Res* **22**: 4673–4680.
31. Thorn KS, Bogan AA. 2001. ASEdb: a database of alanine mutations and their effects on the free energy of binding in protein interactions. *Bioinformatics* **17**: 284–285.
32. Tsai CJ, Lin SL, Wolfson HJ, Nussinov R. 1997. Studies of protein–protein interfaces: a statistical analysis of the hydrophobic effect. *Protein Sci* **6**: 53–64.
33. Valdar WS. 2002. Scoring residue conservation. *Proteins* **48**: 227–241.
34. Valdar WS, Thornton JM. 2001. Conservation helps to identify biologically relevant crystal contacts. *J Mol Biol* **313**: 399–416.
35. Xenarios I, Salwinski L, Duan XJ, *et al*. 2002. DIP, the Database of Interacting Proteins: a research tool for studying cellular networks of protein interactions. *Nucleic Acids Res* **30**: 303–305.
36. Yao H, Kristensen DM, Mihalek I, *et al*. 2003. An accurate, sensitive, and scalable method to identify functional sites in protein structures. *J Mol Biol* **326**: 255–261.
37. Zarembinski TI, Hung LW, Mueller-Dieckmann HJ, *et al*. 1998. Structure-based assignment of the biochemical function of a hypothetical protein: a test case of structural genomics. *Proc Natl Acad Sci USA* **95**: 15 189–15 193.

Copyright © 2003 John Wiley & Sons, Ltd. 
